# Mechanisms of Homoarginine: Looking Beyond Clinical Outcomes

**DOI:** 10.1111/apha.14273

**Published:** 2025-01-16

**Authors:** Ashley Zubkowski, Amanda N. Sferruzzi‐Perri, David S. Wishart

**Affiliations:** ^1^ Department of Biological Sciences University of Alberta Edmonton Alberta Canada; ^2^ Centre for Trophoblast Research, Department of Physiology, Development and Neuroscience University of Cambridge Cambridge UK; ^3^ Department of Computer Sciences University of Alberta Edmonton Alberta Canada

**Keywords:** biomarker, cardiovascular, mechanisms, metabolite, pathways, pregnancy

## Abstract

**Purpose:**

Homoarginine (hArg) is an arginine metabolite that has been known for years, but its physiological role in the body remains poorly understood. For instance, it is well known that high hArg concentrations in the blood are protective against several disease states, yet the mechanisms behind these health benefits are unclear. This review compiles what is known about hArg, namely its synthetic pathways, its role in different diseases and conditions, and its proposed mechanisms of action in humans and experimental animals.

**Findings:**

Previous work has identified multiple pathways that control hArg synthesis and degradation in the body. Furthermore, endogenous hArg can modulate the cardiovascular system, with decreased hArg being associated with cardiovascular complications and increased mortality. Studies also suggest that hArg could serve as a diagnostic biomarker for a variety of immune, pancreatic, renal, and hepatic dysfunctions. Finally, in women, hArg concentrations rapidly increase throughout pregnancy and there are suggestions that alterations in hArg could indicate pregnancy complications like pre‐eclampsia.

**Summary:**

Homoarginine is an under‐appreciated amino acid with potential wide‐ranging roles in systemic health, pregnancy, and pathophysiology. Although recent research has focused on its health or disease associations, there is a need for more investigations into understanding the mechanistic pathways by which hArg may operate. This could be aided using metabolomics, which provides a comprehensive approach to correlating multiple metabolites and metabolic pathways with physiological effects. Increasing our knowledge of hArg's roles in the body could pave the way for its routine use as both a diagnostic and therapeutic molecule.

## 1 INTRODUCTION

L‐Homoarginine (hArg) (N6‐(aminoiminomethyl)‐L‐lysine) is an amino acid homologue of L‐Arginine (Arg). Compared to Arg (C_6_H_14_N_4_O_2_), hArg (C_7_H_16_N_4_O_2_) has an almost identical chemical structure but it has an additional methylene inserted into its sidechain. While Arg is a proteogenic, conditionally essential amino acid that plays key roles in infant/child development, nitric oxide (NO) synthesis and recovery from trauma,^1^, ^2^, ^3^, ^4^ hArg, on the other hand, is a non‐essential, non‐proteogenic amino acid. While initially thought to be diet‐derived (via plant foods), evidence that hArg could be endogenously produced by mammals first emerged as early as 1963.^5^ This discovery coincided with an increase in published work on hArg in the 1960s and 1970s that continues even today. However, for many years after this initial discovery, hArg was still believed by many scientists to be a low abundance, diet‐derived compound with no biological relevance. As a result, hArg was often used as an “exogenous” calibration standard in high‐performance liquid chromatography (HPLC) to measure other methylated arginine metabolites, such as asymmetric dimethylarginine (ADMA) and symmetric dimethylarginine (SDMA) in humans or other mammals.^6^ However, over the years, this perception that hArg was an irrelevant dietary contaminant has been shown to be mistaken and hArg has been consistently found in all mammals, in most biological fluids (namely serum, urine, cerebral spinal fluid) and most tissues (including brain, liver, and kidney), although at low concentrations.^7^, ^8^, ^9^, ^10^, ^11^, ^12^ For instance, a healthy human adult has typical hArg blood concentrations in the range of 2.5 ± 1.0 μM, whereas for Arg, the blood concentrations range from 77.4 ± 18.2 μM.^13^, ^14^

**FIGURE 1:**
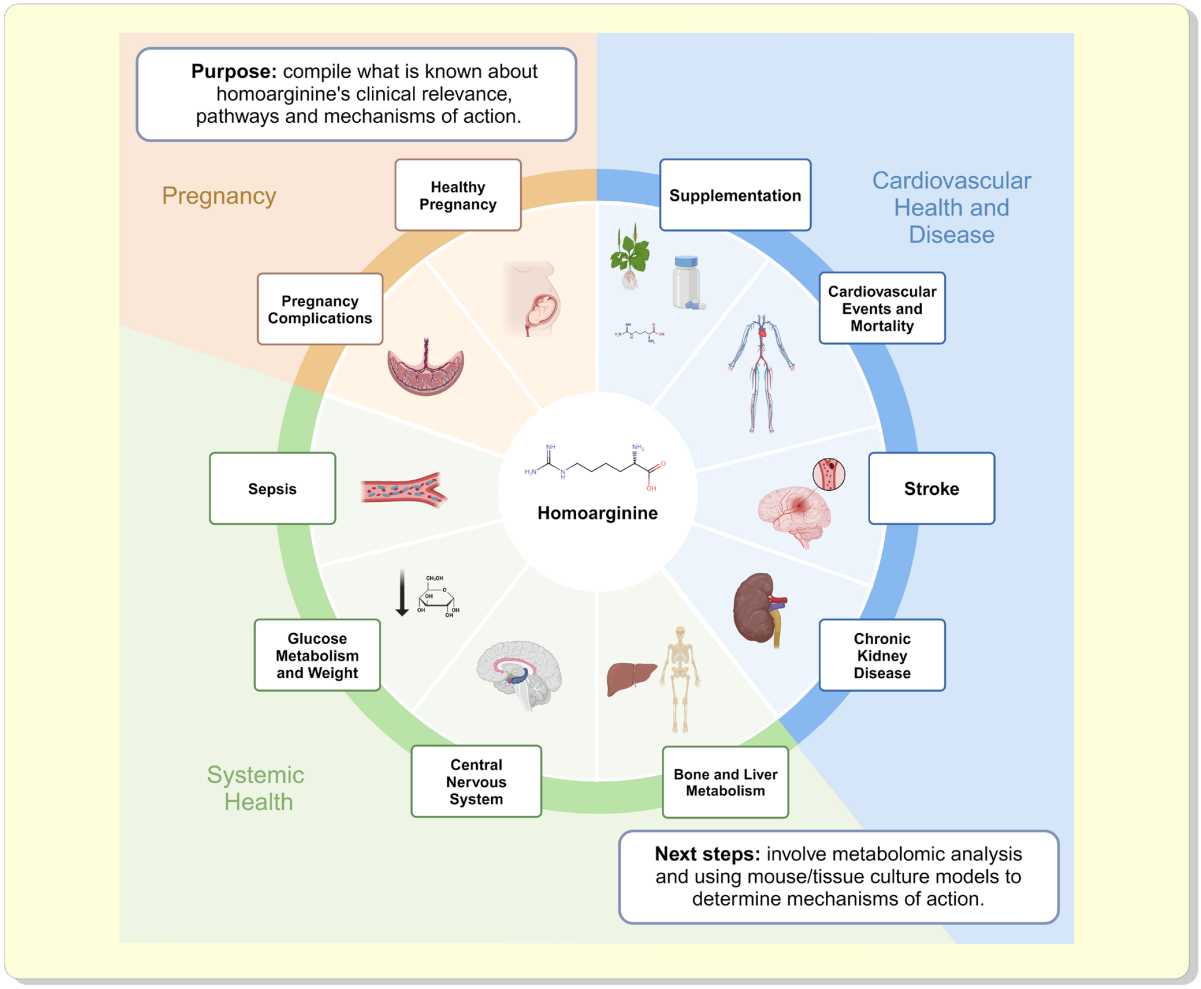
Homoarginine (hArg) is clinically relevant in many fields, including cardiovascular health and disease, systemic health, and pregnancy. HArg has various roles in both the progression of healthy pregnancies and pathology of pregnancy complications. Systemically, hArg is associated with changes in glucose metabolism and weight regulation, the central nervous system, bone and liver metabolism, and sepsis severity. In terms of cardiovascular health, hArg is linked to supplementation outcomes, cardiovascular events, mortality rates, stroke severity, and chronic kidney disease. Created in BioRender. Wishart, D (2024). BioRender.com/q08i861 (#BI27EQM52M).

The fact that hArg is found in all mammals and the fact that it is endogenously produced, led to more concerted studies in the 2010s exploring why this particular Arg analog is so conserved across species and what it is actually doing in the body. As a result of these investigations, a number of interesting associations between hArg and health have been found. For instance, many studies have shown that higher serum/plasma concentrations of hArg are positively associated with cardiovascular health, while lower concentrations of hArg tend to indicate cardiovascular complications.^7^, ^15^, ^16^ HArg has also been indicated as a potential biomarker for numerous clinical conditions or diseases. These include pregnancy and pregnancy‐associated complications, renal and parathyroid diseases, type 1 and 2 diabetes and various inflammatory conditions. Rather than focusing on the endogenous effects or clinical outcomes associated with homoarginine, as done with other reviews,^10^, ^17^, ^18^ this review aims to provide a detailed overview of the role of hArg in the body by describing hArg's metabolism, its biosynthetic pathways and its presumptive roles and mechanisms of action in human/animal physiology and disease (Figure 1). This review will also highlight the need for more directed research to understand how hArg may function and how a better understanding of its mechanisms may be informative for improving clinical outcomes.

### 1.1 Homoarginine Biosynthesis and Metabolism

**FIGURE 2:**
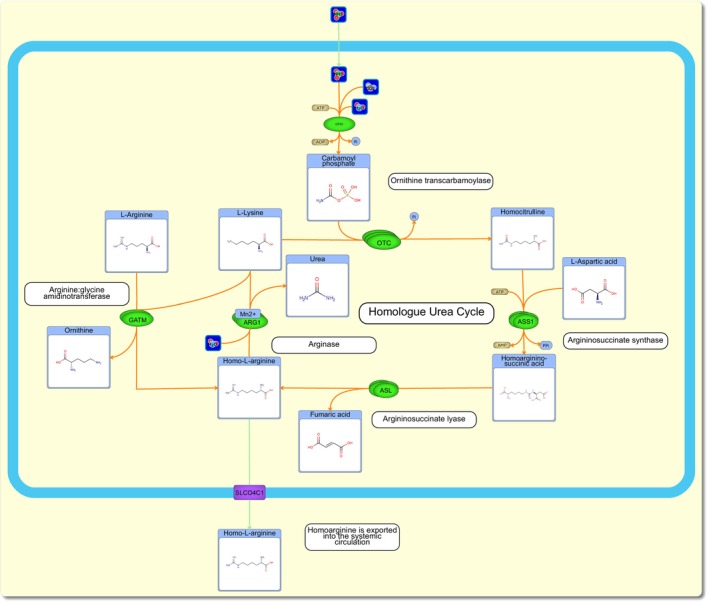
Metabolic pathway of homoarginine (hArg) synthesis and associated substrates. Nitric oxide enters cells by diffusion where its products interact with multiple enzymes (CPS1, carbamoyl phosphate synthetase 1; ASS1, argininosuccinate synthase; ASL, argininosuccinate lyase) in a homologue urea cycle including ornithine transcarbamoylase (OTC) to create hArg. Arginase (ARG1) can then breakdown hArg into lysine. HArg is also synthesized from lysine and Arg by the arginine: glycine amidinotransferase (AGAT) enzyme. Figure created on PathBank 2.0.^23^

In mammals, hArg is synthesized in the liver, brain, small intestine and kidney and released into many biological fluids^19^, ^20^, ^21^ via the cationic amino acid transporters CAT1, CAT2A and CAT2B.^22^ HArg can be synthesized either from Arg through the arginine: glycine amidinotransferase (AGAT) enzyme or from lysine through the ornithine transcarbamoylase (OTC) enzyme. AGAT catalyzes glycine and Arg to guanidino acetic acid (GAA) and ornithine (Orn) in the creatine biosynthesis pathway (Figure 2). Synthesis of creatine is primarily done in the kidney, where the AGAT enzyme is highly expressed. AGAT can also use lysine instead of glycine with Arg and produce Orn and hArg instead of GAA.^20^ AGAT has been physiologically confirmed to synthesize hArg, as AGAT^−^/^−^ mice have significantly lower hArg plasma concentrations than wild‐type mice.^24^, ^25^ When the guanidinoacetate N‐methyltransferase gene is deleted in mice (Gamt^−^/^−^) an upregulation of AGAT occurs and hArg plasma concentrations increase.^26^ In the human genome, a single‐nucleotide polymorphism (SNP) at chromosome 15 for AGAT (GATM: EC 2.1.4.1) has been strongly associated with serum hArg concentrations.^26^, ^27^ Although AGAT is known to synthesize hArg, its affinity for lysine (which is needed for hArg synthesis) is much lower than for glycine (which is needed for GAA synthesis). Understanding alterations to this substrate affinity has helped improve our understanding of how and why hArg concentrations can vary under different physiological states.

In addition to the role of AGAT in hArg production, the OTC enzyme has also been shown to catalyze the formation of hArg from lysine in a homologous form of the urea acid cycle (Figure 2). Although OTC has a strong affinity for Orn and carbamoyl‐phosphate, which leads to the production of citrulline, lysine can also be used as an alternative substrate and will be converted by OTC to homocitrulline.^28^, ^29^ Homocitrulline (hCit) and aspartate undergo a condensation reaction via argininosuccinate synthetase (ASS) into homoarginino‐succinate (ASA). ASA is then separated into hArg and fumarate by the enzyme known as argininosuccinase. A gene on the carbamoyl phosphate synthetase I (CPS1) enzyme (EC 2.6.1.44) has been identified as a strong predictor of hArg serum concentrations.^26^, ^27^ This is expected as CPS1 is the rate‐limiting enzyme in the urea cycle and produces carbamoyl‐phosphate, which is essential for the function of the OTC enzyme. If carbamoyl‐phosphate is in limited supply, the entire urea acid cycle (and its homologous cycle) will arrest. HCit can also be formed through a spontaneous process called carbamylation, which involves a non‐enzymatic modification of lysine by a urea‐derived cyanate.^30^ Synthesis of hCit is known to lead to an increase in homologous urea cycle activity.^29^

HArg has also been shown to be hydrolyzed by arginase to form urea, and genetic variations in the arginase (MED23/ARG1) locus on chromosome 6 have also been associated with variations in serum hArg levels.^19^, ^27^ This route of hArg degradation and hArg regulation will be discussed further below. A better‐understood route for hArg degradation has been found in the mitochondrial enzyme alanine: glyoxylate aminotransferase 2 (AGXT2). In the absence of AGXT2, in AGXT2 knockout mice, hArg was found to accumulate in plasma.^31^ After being transported into the mitochondria by the ORC1, ORC2, and SLC25A29 transporters, hArg is metabolized by AGXT2 to 6‐guanidino‐2‐oxocaproic acid (GOCA) and is believed to be involved in human and mouse hArg homeostasis. GOCA concentrations in plasma are reported to be much lower than hArg concentrations.^32^ The AGXT2 locus (EC 2.6.1.44) has also been associated with changes in hArg serum concentrations.^27^

### 1.2 Homoarginine Signaling and Mechanisms of Action

While the synthesis and metabolism of hArg are generally well understood, its roles in molecular signaling and its mechanisms of action are less well understood. To date, there is good evidence that hArg can modulate nitric oxide (NO) production through its interaction with NO synthase (NOS).^33^, ^34^ Additionally, it is known that hArg can modulate levels of Arg and Orn by inhibiting the action of arginase,^34^ that hArg can increase *Kcnip3* and *Ivd* gene expression,^35^ that hArg binds to and inhibits bone and liver alkaline phosphatase^36^ and that hArg can bind to Myh9 (myosin heavy chain 9), which inhibits T‐cell activation and proliferation.^37^

The role of hArg in NO production is perhaps best known and best understood. NO plays a significant role in controlling hemodynamic changes, specifically increasing vasodilation and blood volume.^38^, ^39^ NOS normally utilizes the rate‐limiting substrate Arg to synthesize NO and L‐citrulline but can also be influenced by Arg analogs. HArg is involved with NO in multiple ways by: (1) acting in competition with Arg as an activating substrate for NOS to release NO during vasodilation and (2) acting as a weak inhibitor of the arginase enzyme.^34^ In both situations, hArg increases the local concentrations of Arg and NO. Increased concentrations of Arg are then present to produce further NO. When hArg is a substrate for NOS, it is converted into hCit.^40^ Activation of NOS also indicates hArg's role in various body systems due to the three NOS isoforms: neuronal, inducible, and endothelial.^26^, ^38^ All three NOS isoforms exhibited a similar mRNA expression level in AGAT^−^/^−^ and wildtype mice.^26^ In one study by Büttner et al., obese ZSF1 rats had a significant deficiency of hArg that was rescued with hArg supplementation before and after heart failure characteristics progressed.^41^ Both eNOS protein and gene expression from aorta tissues remained unchanged in all groups, but aorta tissue iNOS was higher in obese compared to lean rats. Measurements for iNOS protein and gene expression were conducted in liver, kidney, skeletal muscle, and small intestine tissues with varying changes in each after hArg supplementation. The expression of iNOS was sensitive to hArg supplementation, but the extent and direction were found to vary systemically.

The modulation of Arg and Orn levels in the body appears to be (partially) done by hArg inhibiting the action of arginase. Arginase has a contradictory relationship with hArg. Previous studies have indicated that hArg is a substrate for arginase, but not an inhibitor, while other studies suggest hArg is an inhibitor without being a substrate.^34^, ^42^, ^43^, ^44^ A study by Tommasi et al.^34^ used cells expressing recombinant arginase 1 and 2 to determine if hArg can inhibit the enzyme's conversion of Arg to Orn. At human physiological serum concentrations (1–10 μM), only 10% of arginase activity was inhibited, but at high hArg concentrations (2–3 mM) arginase activity was significantly inhibited. HArg was not detected as a substrate for arginase 1 or 2 in their experimental conditions. Arginase may assist with the disposal of hArg, but the complicated relationship between hArg and arginase requires further study.

The role of hArg in regulating *Kcnip3* and *Ivd* gene, as well as KNIP3 and IVD protein expression is a relatively recent finding.^35^ KCNIP3, also known as calsenilin, is a neuronal calcium‐binding protein that has been shown to have multiple cellular functions, including regulating the intracellular concentration of calcium, binding and modulating presenilin (an Alzheimer's‐disease‐related protein) and controlling multiple signaling pathways as a second messenger.^35^, ^45^ The IVD enzyme, also known as Isovaleryl‐CoA dehydrogenase, plays a role in L‐leucine degradation. It is also notable that branched‐chain amino acids, particularly leucine, play an important role in glutamate synthesis and glutamate is an important neurotransmitter. While hArg supplementation can normalize *Ivd* gene expression levels in the brain tissue of AGAT^−^/^−^ mice, leucine concentrations did not normalize with hArg supplementation.^35^

In contrast to the recent discovery of hArg regulating *Kcnip3* and *Ivd* gene expression, the discovery that hArg binds and inhibits bone and liver alkaline phosphatase (APs) dates from 1972.^36^ Other amino acids, such as phenylalanine and tryptophan, are known to inhibit placental and intestinal APs but hArg does not inhibit these enzymes in human tissue. Likewise, phenylalanine and tryptophan cannot inhibit liver or bone APs. The specificity of hArg for these liver and bone APs has been shown to lead to increased bone density and decreased bile secretions.^46^ In contrast, a study by Suzuki et al.^47^ determined that bovine or porcine intestinal, placental and kidney APs were inhibited by hArg. In agreement with Lin and Fishman,^36^ hArg was found to inhibit bone APs from an osteogenic mouse cell line. It was determined that the differences in hArg binding capabilities may be due to the number of binding sites on each AP isoenzyme. One study, by Aghdassi et al.^48^ also found that serum hArg concentrations had an inverse association with blood AP concentrations, but only in men and post‐menopausal women. Pre‐menopausal women did not show any association between hArg and AP concentrations. These conflicting results suggest that hArg may interact with APs in a very specific manner depending on species, isoenzymes, and hormone levels.

The recent finding that hArg binds to Myh9 as reported by Nitz et al.^37^ is particularly exciting as it appears to help explain hArg's immunosuppressive activity as well as some of its anti‐atherogenic effects. *Myh9* gene expression was previously found to be normalized in AGAT^−^/^−^ mice brains after oral supplementation of hArg.^35^ Nitz et al. subsequently determined that when hArg binds to Myh9, it inhibits myosin regulatory light chain phosphorylation and profoundly modulates the spatial organization of the T‐cell actin cytoskeleton.^37^ This increases T‐cell filopodia formation which, in turn, impairs actin‐dependent functions such as T‐cell activation, mobility, migration (in response to relevant chemokines), and proliferation.^37^ This immunosuppressive activity, which helps prevent atherosclerotic plaque formation, appears to explain hArg's atheroprotective effects.

No doubt other hArg signaling mechanisms still remain to be discovered and given the widespread, pleiotropic effects of hArg on mammalian physiology, it is likely that many more pathways, molecular targets and molecular mechanisms of action will be revealed in the coming years.

### 1.3 Measurement of Homoarginine

Many of the earliest studies that detected hArg in biological fluids utilized high‐performance liquid chromatography (HPLC) coupled with fluorescence detection. This was made possible after the derivatization of hArg with a phenanthrenequinone fluorophore in alkali solutions.^9^, ^20^ More recent studies have used liquid chromatography–mass spectrometry (LC–MS) for hArg detection and quantification. LC–MS offers greater sensitivity, better separation from other molecules (via retention time and mass), easier work‐up and, in many circumstances, allows more accurate quantification via the use of isotopically labeled versions of hArg as reference standards.^25^, ^49^, ^50^ The greater sensitivity allows this specific quantification method to not only detect homoarginine in biological fluids at low concentrations (< 0.1 μM), but is used in metabolomics studies with the measurement of hundreds of other compounds. Other approaches to hArg detection have used gas chromatography–mass spectrometry (GC–MS), which provides similar advantages in terms of sensitivity and quantification as LC–MS.^51^, ^52^ Most studies that detect multiple compounds (for example, compounds such as hArg, Arg, and ADMA) will utilize metabolomics methods and MS quantification. A few hArg enzyme‐linked immunosorbent assay (ELISA) kits (ImmuSmol, Eagle Biosciences) have also been developed commercially, and these report good sensitivity and specificity. Currently, LC–MS and ELISA assays are the most common methods for measuring hArg in the plasma of humans and other mammals, although reported values of hArg tend to be higher in LC–MS measurements when directly compared to ELISA or HPLC methods.^25^

## 2 HOMOARGININE IN CARDIOVASCULAR HEALTH AND DISEASE

Reference concentrations for hArg in serum are generally listed at 2.5 ± 1.0 μmol/L (mean ± standard deviation) with serum concentrations of hArg tending to be higher in men than women.^53^, ^54^, ^55^ Regardless of sex, hArg concentrations have been shown to decline with age.^13^, ^49^ Low serum and plasma levels of hArg are generally associated with higher risk of cardiovascular disease.^56^ On the other hand, elevated levels of another Arg metabolite, ADMA, are typically associated with an increased risk of cardiovascular disease.^57^ Therefore, the ratio between hArg and ADMA can be quite diagnostically informative, with low hArg to ADMA ratios typically being associated with poor cardiovascular health or outcomes and high hArg to ADMA ratios being associated with better cardiovascular health or outcomes.^58^ One note of caution is that dietary intake of some legumes, such as grass pea (*Lathyrus sativus L*.), can increase hArg concentrations in the blood.^59^ In addition, the repeated consumption of high amounts of nitrate, hArg and, citrulline can increase circulating hArg.^52^, ^60^, ^61^ Other foods that contain high concentrations of these metabolites, such as beets and watermelon when consumed in high quantities, could alter measured blood hArg levels. Therefore, in an ideal hArg study, dietary factors should be considered to remove any potential confounding results. While the number of clinical studies associating hArg levels with cardiovascular disease is in the hundreds, few of these studies have proposed any mechanisms. Interestingly, one study by Hou et al.^8^ found that after one dose of hArg supplementation in pigs and rats, 95%–96% of ingested hArg was excreted in their urine. Further work is needed to improve our mechanistic understanding of hArg and the diagnostic/biomarker potential of quantifying its concentrations in the blood and urine in humans and other mammalian species.

### 2.1 Supplementation

HArg has been proposed to be a cardioprotective metabolite due to its positive association with improved cardiovascular outcomes, and lower cardiovascular mortality rates.^18^, ^55^, ^62^ Therefore, supplementation with hArg to protect against cardiovascular disease has been the subject of several clinical trials. One such phase 1 trial involved giving 20 healthy volunteers 125 mg hArg daily for 4 weeks.^60^ This dose increased plasma hArg concentrations and was found to be safe, had no toxic side effects, and was well tolerated by participants of the trial. Phase 2 of the study is currently underway and is looking at normalizing hArg blood concentrations with oral supplementation in patients with acute ischemic stroke (URL: https://clinicaltrials.gov. Unique identifier: NCT03692234). A much cheaper supplement that appears to increase hArg levels is inorganic nitrate. Specifically, inorganic nitrate supplementation in healthy men has recently been shown to shift AGAT to biosynthesize hArg over GAA (Figure 2).^52^ Of particular interest, this study used GC–MS to measure 27 other metabolites, including amino acids, creatinine, and oxidative stress markers. By measuring multiple metabolites in the AGAT pathway, these authors were able to determine the concentration changes of each metabolite in the AGAT synthesis pathways. This comprehensive data confirmed that the equilibrium of biosynthesis in the AGAT enzyme shifted towards using lysine for hArg synthesis over utilizing glycine to form GAA, but oxidative stress levels did not change after supplementation with inorganic nitrate. Overall, hArg and inorganic nitrate supplementation may be a safe option to protect heart health in individuals without complications.

In AGAT^−^/^−^ mouse models with diseases, such as stroke, heart failure and muscle damage, the impact of hArg supplementation differs. A suppression of AGAT gene expression in mice and humans has been associated with statin‐induced myopathy.^63^, ^64^, ^65^ Myopathy is a disease impacting muscle fiber function and pain that can start after taking statins, a medication used to reduce the risk of heart disease and lower cholesterol. Sasani et al. found that cerebrovascular patients treated with statins had lower hArg plasma concentration than patients without.^65^ This study also utilized hArg‐supplemented AGAT^−^/^−^ mice with simvastatin‐induced muscle damage but did not see an improvement. Although hArg‐deficient AGAT^−^/^−^ mice had reduced muscle strength after simvastatin treatment, hArg supplementation was seen to have no effect. In contrast, other studies have found that supplementing hArg in AGAT^−^/^−^ mice with induced vascular pathologies resulted in improved or normalized outcomes.^26^, ^66^ Thus, the type and severity of cardiovascular pathology, as well as dose and length of hArg supplementation may be critical for determining whether hArg would offer any benefit or not.

### 2.2 Cardiovascular Events and Mortality

HArg is thought to regulate cardiac output during rest and exercise. In older individuals (aged 50–87 years), a positive association was found between plasma hArg and systolic/diastolic blood pressure.^67^ While higher blood pressure can be detrimental to health, this cardiovascular change indicates that hArg is a putative regulator of cardiovascular function. Other work has shown that in an extreme circumstance of diminished cardiac function, both mice and human patients with heart failure were found to have lower serum hArg.^68^, ^69^ Lower hArg values also predicted a more severe form of heart failure along with higher levels of other heart failure markers including N‐terminal pro b‐type natriuretic peptide.^68^ Low serum hArg concentrations have also been associated with increased levels of markers for endothelial dysfunction while high hArg concentrations are associated with the inhibition of platelet aggregation through diminished levels of fibrinogen and d‐dimer.^62^, ^70^ Furthermore, for patients with heart failure, those with increased hArg concentrations in their blood prior to exercise showed a greater improvement in peak VO_2_ levels, a measure of exercise capacity.^71^

Diminished hArg concentrations in the blood have been associated with increased adverse cardiovascular events and all‐cause mortality in many other studies. An investigation by März et al.^62^ found that decreased levels of serum hArg were associated with increased cardiovascular death rates in patients referred for coronary angiography (7‐year follow‐up) and diabetic patients undergoing hemodialysis (4‐year follow‐up). Individuals with lower hArg also had a 2.7× higher all‐cause mortality. Likewise, a study in a black South African population (10‐year follow‐up) found that lower plasma hArg levels were associated with an increased risk of 10‐year cardiovascular and all‐cause mortality.^72^ Higher rates of mortality in individuals with decreased serum hArg concentrations have been found in other studies from primarily white^55^, ^73^, ^74^ and mixed ethnicity^54^ cohorts. In a meta‐analysis review of 13 studies, high circulating hArg concentrations were inversely associated with all‐cause mortality, regardless of sample type (serum vs. plasma) or detection method (MS vs. fluorescent).^17^ This reinforces that hArg is not just another amino acid. Other work has shown that individuals exposed to tobacco smoke had significantly lower (17%) plasma hArg concentrations when compared to non‐smokers.^15^ This is interesting given it is well established that cigarette smoking negatively impacts cardiovascular health and increases the risk of all‐cause mortality. Although not all causes of mortality may be directly linked to low levels of hArg, evidence suggests that higher plasma/serum hArg levels are better for overall health.

### 2.3 Stroke

As cerebrovascular diseases rely heavily on the proper functioning of both neuronal NOS and endothelial NOS, hArg could be a useful biomarker for complications such as a stroke.^75^, ^76^, ^77^ In humans, plasma hArg concentrations were higher in patients who survived 7 years after an ischemic stroke or did not have an additional event within 30 days post‐stroke than in individuals who did not survive.^26^ In the same study, knockout (Agat ^−^/^−^) mice (i.e., those with little or no endogenous hArg) were found to have more extensive cerebral damage and more neurological defects after being subjected to experimental strokes. Severe incidences were reduced in the knockout mice supplemented with hArg.^26^ In contrast, mice with increased expression of AGAT (Gamt^−^/^−^), and therefore high levels of hArg, when subjected to an experimental acute ischemic stroke, were found to have a diminished infarct size compared to controls. Taken together, these data suggest that high hArg levels and/or AGAT upregulation may attenuate stroke severity and predict positive patient outcomes post‐stroke in both human and mouse models.

In addition to stroke outcomes, hArg can help distinguish stroke subtypes and associated symptoms. Post‐ischemic stroke and transient ischemic attacks are associated with aortic intima‐media thickness and aortic distensibility during aortic wall remodeling. In humans, a high plasma hArg/ADMA ratio or low plasma hArg alone was strongly associated with aortic intima‐media thickness or aortic atherosclerosis but not aortic distensibility.^78^ Interestingly, these effects were more pronounced in males. Further, Cordts et al.^79^ determined various stroke subtypes, risk of stroke recurrence and predicted internal carotid artery stenosis/occlusion using plasma hArg/ADMA and hArg/SDMA ratios. These ratios could distinguish small vessel diseases from large vessel diseases and cardio‐embolisms (lower hArg/ADMA ratios). Patients with atrial fibrillation also exhibited lower hArg/ADMA ratios than patients without.^79^ These authors also reported that a lower hArg/SDMA ratio was associated with a higher risk of stroke recurrence. Given that limited differences were found for ADMA and SDMA only, it appears that hArg is likely the primary driver in stroke occurrence.

### 2.4 Chronic Kidney Disease

Given the frequent co‐occurrence of chronic kidney disease (CKD) with cardiovascular disease, it is not surprising that studies measuring hArg levels in CKD have also been conducted. One study involving 527 patients with CKD found that lower plasma hArg levels indicated more severe renal functional impairments and a poorer prognosis in renal and cardiovascular endpoints (kidney and heart replacement or disease).^32^ This study also demonstrated that higher GOCA concentrations were accompanied by reduced plasma hArg levels, which together suggest elevated AGXT2 levels are present (the main hArg degradation enzyme) in those with severe CKD.^32^ Likewise, lower GOCA concentrations and, by association, higher hArg levels, were associated with better cardiovascular function, as well as better renal function. In addition, one study found that CKD patients had incrementally lower plasma hArg with diminished glomerular filtration rate.^80^ Individuals who had renal disease progression in a follow‐up of up to 7 years were found to have significantly lower hArg at baseline than individuals whose kidney disease did not progress. A few metabolomic studies have identified carbamylation and the production of hCit, another metabolite in the homologous urea acid cycle, as a marker for CKD progression^81^ and have verified its performance against other markers.^82^, ^83^ Given the close relationship between hCit and hArg, hArg's excretion in the urine,^8^ and hArg's association with both glomerular filtration rate and CKD progression, more extensive studies should be performed to help determine if hArg could serve as a CKD biomarker and uncover the underlying mechanistic pathways.

## 3 HOMOARGININE IS ESSENTIAL FOR SYSTEMIC HEALTH

Many hArg studies focus on cardiovascular health and disease, but hArg is not just another cardiovascular metabolite. The enzymes introduced in Sections 1.1 and 1.2 function in tissues all over the body, indicating a broad, systemic role for hArg. These fields of research successfully utilize animal models and metabolomics to expand upon mechanistic insights unattainable in clinical studies. The following sections discuss these systemic roles for a number of conditions in more detail.

### 3.1 Sepsis

Over the past 10 years, several studies have analyzed the role of hArg in predicting sepsis onset and severity. During sepsis, increased NO production is essential for increased vasodilation, which is needed for sufficient oxygenation of tissues and organs to avoid septic shock.^84^, ^85^ As NO is difficult to measure directly in plasma/serum due to its short half‐life, many studies utilize NO substrates (ADMA, Arg, and hArg) for indirect measurements. In a 2017 study by Winkler et al.^84^ plasma samples of 100 septic patients were taken within 24 h of admission to an intensive care unit (ICU). These samples were analyzed by LC–MS to measure ADMA, Arg, and hArg levels. Patients with either surgical trauma (hArg = 1.06 μmol/L), sepsis (hArg = 0.92 μmol/L), or septic shock (hArg = 0.79 μmol/L) had significantly lower plasma hArg concentrations compared to healthy controls, which averaged 1.89 μmol/L. The more severe the diagnosis, the lower the hArg concentration. A low hArg:ADMA ratio was also correlated with septic shock organ failure. Later studies were able to use hArg to differentiate adult patients with sepsis from healthy and infected patients without sepsis as well as neonate patients with sepsis from neonates with meningoencephalitis.^86^, ^87^ HArg has also been found to predict sepsis of varying degrees in adults and newborns, the length of hospital/ICU stay and onset of worsening sepsis reactions.^86^ These data suggest hArg could be a very useful clinical biomarker for monitoring, assessing, or predicting sepsis outcomes.

### 3.2 Glucose Metabolism and Weight

Increased serum hArg concentrations have been shown to predict hyperglycemia and abdominal obesity in men, and type 2 diabetes in women.^88^ Furthermore, the stress hyperglycemia ratio, a reflection of the acute blood glucose change in response to illness, has been found to be positively associated with plasma hArg levels.^89^ On the other hand, evidence indicates that body mass index, weight loss and oral glucose tolerance are not related to changes in hArg concentrations.^90^ The study by May et al.^90^ also found that leaner individuals had higher interstitial adipose hArg levels than individuals who were morbidly obese. Interestingly, weight loss did not alter the concentrations of hArg in serum or adipose tissue. Thus, studies in human cohorts have not been able to properly distinguish hArg as a biomarker for diabetes but suggest that hArg could be involved in the development, or be a consequence, of the disease.

Studies in experimental animal models may help shed more light on the role of hArg in diabetes‐associated conditions. For instance, Stockebrand et al.^91^ supplemented C57BL/6J control mice and high‐fat diet mice with hArg and found that after 8 weeks of a high‐fat diet, hArg dose‐dependently improved fasting blood glucose concentrations. This was independent of a change in weight, glucagon concentration and insulin tolerance responses to the diet. Increased fasting insulin concentrations were detected only with high‐dose hArg supplementation. This study concluded that a glucose‐lowering effect of hArg occurs in diet‐induced obese mice and that hArg may offer some benefit and compensate for the glucose intolerance of type 2 diabetic patients. Both Arg and hArg have been shown to dose‐dependently induce the release of glucagon and insulin in isolated mouse pancreatic islet cells. This provides further evidence of the importance of Arg and hArg in glucose regulation.^92^ A study by Wetzel et al.^11^ utilized mice with induced diabetic nephropathy (*Ins2* ^*Akita*^), a disease that damages the kidney typically due to hypertension, to study hArg supplementation as a potential treatment for type 1 diabetic patients.^11^ Mice with diabetic nephropathy had reduced kidney tissue levels of hArg, Arg, and NO compared to wild‐type littermates. Supplementation with hArg for 12 weeks did not alter the body weight or blood glucose of the diabetic mice. Still, hArg supplementation reduced albuminuria and renal histological changes, glomerular macrophage infiltration and oxidative stress markers while increasing nitrite production in the kidneys of diabetic mice. Although human studies did not identify hArg as an overall diagnostic marker of diabetes, diabetic animal model studies have found pancreatic and kidney specific changes in hArg concentrations that reflect a number of associations found in human cohort studies.

### 3.3 Central Nervous System

Although multiple enzymes associated with hArg are present in human brain tissue, there is remarkably little information on how hArg functions in either healthy or diseased brains. The presence of AGAT and OTC in the brains of both humans and rats indicates the potential for local synthesis of hArg.^10^ Cationic amino acid transporters (hCATS) are known to be located in human/rat brains, which allows for hArg uptake along with removal and transport within brain regions.^10^, ^22^ Oral supplementation with hArg has been shown to regulate the expression levels of *Kcnip3* mRNA in the brain tissue of AGAT^−^/^−^ mice.^35^ Mice with diminished *Kcnip3* typically present with severe cognitive impairment. In a small study of 20 human participants, supplementation with hArg daily for 4 weeks neither improved or impaired cognitive performance or brain health.^93^ The effects of long‐term hArg supplementation on cognitive performance have not been studied.

### 3.4 Bone and Liver Metabolism

Increased serum/plasma levels of hArg have been shown to inhibit bone and hepatic alkaline phosphatases (APs), leading to increased bone density and decreased bile secretions.^46^, ^48^, ^94^, ^95^ Low hArg is associated with higher bone turnover, higher parathyroid hormone (PTH) concentrations and lower bone density.^94^, ^95^, ^96^, ^97^ Of note, the study by Tomaschitz et al.^96^ determined that patients with primary hyperparathyroidism (PHPT) who have an excess PTH concentration will typically have lower circulating hArg (1.16 vs. 1.62 mmol/L, respectively) than controls (59 PHPT vs. 92 controls). In another example of metabolomics analysis helping to determine mechanistic pathways of health, the paper by Langen et al.^98^ utilized GC–MS analysis of ADMA, Arg, and hArg concentrations for plasma of 66 children with short stature, with and without growth hormone deficiency, and 24 children with normal growth. The study determined a positive correlation between insulin‐like growth factor 1 and hArg/ADMA ratio in both short‐stature groups. By examining the concentrations of all three metabolites, a mechanism by which ADMA modulates growth was proposed. HArg positively contributes to growth by inhibiting ADMA suppression of NO and promoting insulin‐like growth factor 1 to increase growth hormone secretion.

In the context of hepatic function, Aghdassi et al.^48^ studied the relationship between hArg and liver biomarkers with population studies of 7638 individuals and found a positive association of increased serum hArg and abnormal levels of liver biomarkers (alanine transaminase, albumin, liver fat content, aspartate aminotransferase, and cholinesterase) in men. While women of pre‐menopausal and post‐menopausal status had limited associations with hArg, with only liver fat content and alanine transaminase, respectively. Both men and post‐menopausal women had inverse associations with AP concentrations. While supplementation of healthy individuals from their previous study^60^ with hArg did not alter liver biomarkers, the elevated hArg level in those with abnormal hepatic function could reflect a compensatory attempt to inhibit AP which would prevent further liver damage and improve bone density. The authors concluded that higher levels of hArg could be a marker for liver dysfunction.

## 4 PREGNANCY

As outlined in Sections 2 and 3, hArg plays many roles throughout the body and in cardiovascular health. Pregnancy involves tightly regulated vasculature changes and numerous systemic changes. While many studies have been conducted on the role of hArg and its associated compounds during both healthy and complicated pregnancies, most of this research is clinical and heavily reliant on association studies. The following section aims to address the lack of mechanistic and metabolomic analysis to better understand hArg's role in pregnancy, but further research is required.

### 4.1 Healthy Pregnancy

Nitric oxide (NO) plays significant roles in the progression of a healthy pregnancy. Indeed, when NO levels are altered, these can be indicative of a number of pregnancy‐related disorders. NO is important for regulating the motility and invasive potential of trophoblasts. Trophoblast regulation is important for recruitment of the maternal blood supply (and hence nutrients and oxygen) to the placenta.^99^, ^100^ NO is also a potent vasodilator, and this is highly relevant during pregnancy as vasodilation supports the dilation of uterine blood vessels and other maternal cardiovascular changes needed for fetal development.^101^ As NO seems to be partially regulated by hArg, interest has increased over the past 20 years in evaluating the role of hArg in controlling the significant hemodynamic changes that occur in the mother's body during pregnancy.

A study by Valtonen et al.^12^ was one of the first to highlight the possible involvement of hArg in human pregnancy (Table 1). Healthy non‐pregnant women (*n* = 61) and pregnant women (*n* = 58) were analyzed for maternal serum hArg by HPLC‐FL. Non‐pregnant women were found to have a hArg concentration of 2.7 ± 1.1 μmol/L, which remained unchanged during the first trimester (3.1 ± 1.4 μmol/L). However, as pregnancy progressed, hArg concentrations increased during the second trimester (4.8 ± 1.7 μmol/L) and continued to increase further during the third trimester (5.3 ± 1.5 μmol/L). The study also found that an increase in flow‐mediated dilation of maternal brachial arteries (a marker for endothelial function) corresponded to an increase in circulating hArg concentrations. In agreement with these findings, expression of the gene encoding the AGAT enzyme is increased by estrogen, a hormone that steadily increases in the circulation of women throughout pregnancy.^104^ As expected, young women taking oral estrogen contraceptives had increased serum hArg concentrations.^88^ A more recent study confirmed these findings with estrogen and determined that individuals taking progestin‐only contraceptives had lower plasma hArg concentrations than non‐users.^105^

**TABLE 1:** Concentrations of homoarginine in serum or plasma during healthy pregnancy.

Reference	Method	Sample	Pregnancy population	hArg concentrations (μmol/L)
Valtonen et al.^12^	HPLC‐FL	Human serum	Nonpregnant, healthy (*n* = 61)	2.7 ± 1.1
			Pregnant, healthy; first trimester (*n* = 13)	3.1 ± 1.4
			Pregnant, healthy; second trimester (*n* = 22)	4.8 ± 1.7
			Pregnant, healthy; third trimester (*n* = 23)	5.3 ± 1.5
Sotgia et al.^102^	LC–MS/MS	Sarda Ewe plasma	Pregnant; day 30 (*n* = 34)	4.7 ± 1.6
			Pregnant; day 60	15.9 ± 7.8
			Pregnant; day 90	173.5 ± 81.6
			Pregnant; day 120	221.4 ± 92.2
			Pregnant; day 150	166.6 ± 53.1
Berlinguer et al.^103^	LC–MS/MS	Sarda Ewe plasma	Pregnant; day 24 (*n* = 59)	~1
			Pregnant; day 50	~30
			Pregnant; day 80	136.1 ± 12.1
			Pregnant; day 120	~110
			Pregnant; day 140	~85
			Postpartum; day 20	~1

Further evidence of hArg's importance in pregnancy can be found by looking at investigations on pregnant sheep. Although placental attachment to the mother in humans and sheep is significantly different, placental vascular formation is similar between these two species. As a result, sheep have been extensively used as human pregnancy models for decades.^106^, ^107^, ^108^ Treatment of non‐pregnant ewes with hArg was shown to improve reproductive physiology through enhanced ovarian angiogenesis, enhanced hormone production and increased folliculogenesis.^109^ A study by Sotgia et al.^102^ found plasma concentrations of hArg to be 100‐fold higher in sheep during mid‐pregnancy than in humans mid‐pregnancy using LC–MS/MS. The large difference in hArg values indicates a potential differentiation of the human discoidal and sheep cotyledonary placentas in how they may use or distribute hArg into their maternal blood. As with humans, hArg concentrations increase significantly during sheep pregnancy. In 34 Sarda breed ewes, hArg baseline concentrations were measured at gestational day 30 (4.70 ± 0.98 μmol/L), rose steadily at day 60 (17.52 ± 7.87 μmol/L), with a further increase at day 90 (171.64 ± 57.84 μmol/L).^102^ Berlinguer et al.^103^ also observed a similar trend in plasma hArg levels during pregnancy in a study of 59 Sarda ewes, although precise concentrations varied from the previous study. In the Berlinguer study,^103^ hArg increased from ~1 μmol/L at day 24 to 136.1 ± 12.1 μmol/L at day 80. In sheep pregnancies, multifold increases in hArg occur between day 24 and day 80 when placental development is extensive and nutrient transport between the mother and fetus is established.^110^ Concentrations of hArg tend to rise during early and mid‐gestation. However, before parturition these levels will steadily decrease and typically return to baseline 20 days after birth.^102^, ^103^ Further work in ewes revealed that hArg changes during pregnancy were not altered by feed restriction. The study by Sotgia et al.^102^ also reported a relationship between significantly increased concentrations of hArg with multiple fetuses (twins and triplets). In pregnancies with twins or triplets, more extensive accommodation on the mother for fetal nutrient demands must occur, and blood flow must increase.^111^, ^112^ Thus, the increase in maternal hArg levels with multiple fetuses is proposed to reflect this increased maternal constraint during multiple gestations.

Whether the increased levels of hArg in maternal circulation reflect production by fetal and placental cells or whether the mother has increased her production of hArg during pregnancy is unclear. Nevertheless, increased levels of circulating hArg would be expected to promote maternal cardiovascular adaptations, as well as changes in the fetal‐placental development and blood flow. Although evidence suggests that hArg has a role in early pregnancy, fetal or placental samples have not been studied. More studies utilizing cell cultures and models locating where this extensive synthesis of hArg is occurring are required to elucidate the significance of hArg in pregnancy.

### 4.2 Pregnancy Complications

Pre‐eclampsia (PE) is a blood pressure condition that develops during pregnancy and is formally diagnosed by the presence of hypertension with proteinuria after 20 weeks of pregnancy. PE can be classified as mild, moderate, or severe, with early or late onset. Although a common disease with high maternal mortality, PE does not have a cure, a treatment, or a complete understanding of its etiology. Previous PE studies have indicated that it is caused by dysregulated uterine spiral artery angiogenesis along with endothelial dysfunction. This is coupled with abnormal placental trophoblast development and invasion, elevated levels of oxidative stress, widespread vasoconstriction, and inflammation.^113^, ^114^ The recent discovery that hArg binds to Myh9 proteins should be further examined due to the associated role of Myh9 in placental development and fetal blood vessel invasion.^115^ Most PE‐associated effects can be directly linked to alterations in the NO pathway. When the NO pathway is disrupted, vascular dysfunction can occur and may cause complications at a time when strictly controlled vasodilation is essential for a healthy pregnancy. In addition, hArg is known to have roles in pregnancy development, endothelial function, vascular development/protection, oxidative stress, and inflammation, indicating why hArg is a topic of interest for studying PE mechanisms.

The first study that measured a change in hArg concentrations in patients with PE was by Khalil et al. in 2011^116^ (Table 2). This study utilized GC–MS to analyze NO pathway analyte concentrations in the plasma of pregnant women at 11–13 weeks of pregnancy. Out of the 375 pregnancies, 75 women developed PE. Out of the 75 PE women, 25 were diagnosed with early‐onset PE, indicating symptoms before 34 weeks of pregnancy and 50 with late‐onset PE (after 34 weeks). Both classifications are a type of PE, but it is accepted that the etiologies of these two forms are different. Early‐onset is typically associated with placentation complications and worse fetal growth outcomes, whereas late‐onset involves dysregulated placental/maternal interactions and maternal predisposition to cardiovascular disease.^119^ Significantly lower hArg and Arg concentrations and higher ADMA/Arg and ADMA/hArg ratios were found in patients with early‐onset PE.^116^ Interestingly, ADMA alone was not significantly different in early‐onset PE. No significant changes in Arg, hArg, or ADMA were found in patients with late‐onset PE. This 2011 study indicated a distinct difference in hArg levels between the two forms of PE and suggested that hArg and other NO‐related metabolites may play a role in the associated trophoblast invasion and placental perfusion complications of early‐onset PE. However, it is important to note that, because the samples were collected early in pregnancy, any indications of late‐onset PE may not be detectable as the placental‐maternal interface is only fully established around the end of the first trimester (12 weeks). Hence, further work is needed.

**TABLE 2:** Concentrations of homoarginine and ADMA in serum or plasma in studies of human pregnancy complications.

Reference	Method	Pregnancy population	hArg concentrations (μmol/L)	ADMA concentrations (μmol/L)
Khalil et al.^116^	GC–MS	Healthy; first trimester (*n* = 300)	3.31 (2.56–4.49)	0.37 (0.34–0.41)
		Preeclampsia; early (*n* = 25)	2.70 (2.30–3.31)	0.38 (0.33–0.45)
		Preeclampsia; late (*n* = 50)	3.53 (2.87–4.74)	0.37 (0.34–0.40)
Yuan et al.^50^	LC–MS/MS	Healthy; <20 weeks (*n* = 17)	5.40 (4.96–6.63)	0.319 ± 0.059
		Mild PE; <20 weeks (*n* = 13)	7.27 (6.87–10.55)	0.347 (0.283–0.354)
		Severe PE; <20 weeks (*n* = 15)	6.27 ± 2.54	0.307 ± 0.044
		Healthy; 20–28 weeks (*n* = 39)	6.07 ± 2.11	0.321 ± 0.039
		Mild PE; 20–28 weeks (*n* = 43)	8.67 ± 2.69	0.354 ± 0.048
		Severe PE; 20–28 weeks (*n* = 39)	7.13 ± 2.88	0.329 (0.305–0.391)
		Healthy; > 28 weeks (*n* = 28)	6.44 ± 2.29	0.380 ± 0.069
		Mild PE; > 28 weeks (*n* = 28)	7.02 ± 2.47	0.418 ± 0.074
		Severe PE; > 28 weeks (*n* = 27)	4.31 (3.48, 5.56)	0.400 (0.354, 0.492)
Speer et al.^117^	HPLC	Healthy; second trimester (*n* = 31)		0.34 ± 0.08
		Healthy; delivery		0.49 ± 0.08
		PE; second trimester (*n* = 15)		0.45 ± 0.09
		PE; delivery		0.55 ± 0.07
		SGA; second trimester (*n* = 12)		0.33 ± 0.06
		SGA; delivery		0.45 ± 0.08
Rizos et al.^118^	ELISA	Healthy; first trimester (*n* = 41)		0.51 ± 0.14
		Healthy; second trimester		0.52 ± 0.13
		Healthy; third trimester		0.58 ± 0.16
		PE; first trimester (*n* = 10)		0.58 ± 0.10
		PE; second trimester		0.63 ± 0.14
		PE; third trimester		0.68 ± 0.11
		SGA; first trimester (*n* = 14)		0.40 ± 0.10
		SGA; second trimester		0.42 ± 0.10
		SGA; third trimester		0.45 ± 0.10

A more recent study separated patients with PE into mild and severe forms and measured hArg and Arg pathway metabolites at various gestational stages.^50^ Serum samples from 84 pregnant women without complications (normo‐tensive controls), 84 with mild preeclampsia, and 81 with severe preeclampsia were collected before 20 weeks, during 20–28 weeks and after 28 weeks of pregnancy. No indication of late versus early diagnosis was reported, but most cases were diagnosed with PE after 34 weeks. All samples were analyzed through LC–MS/MS for Arg, hArg, ADMA, and SDMA concentrations. In contrast to the Khalil et al.^116^ study, hArg and hArg/ADMA were significantly higher before 20 weeks of pregnancy in samples from women with PE than those from healthy controls. Early‐onset PE is more often associated with severe outcomes, while late‐onset is often more mild. In severe PE, ADMA concentrations were not significantly different in the maternal circulation throughout pregnancy, but hArg decreased after 28 weeks.^50^ Samples from mild PE had significantly higher hArg levels in the before‐20 weeks and the 20–28 week group compared to controls but were not higher after 28 weeks. This study concluded that elevated serum hArg could be a potential biomarker for predicting PE.

These two studies involving hArg in PE are difficult to compare directly. Most samples from the Yuan et al.^50^ study were collected later in pregnancy (20+ weeks) than the Khalil et al. study.^116^ Although the study by Yuan et al.^50^ included participants before 20 weeks of pregnancy, data tables indicated a range of 12–17 weeks compared to 11–13 weeks in the Khalil et al.^116^ study. Although there were only a few weeks of difference, week 17 is already in the second trimester when maternal blood flow to the placenta is fully established.^120^, ^121^ Furthermore, as previously mentioned, hArg concentrations rapidly change during this time in human pregnancy. Most individuals with early‐onset PE also delivered before the 28‐week cohort were sampled. The discrepant findings reported in these two PE studies could also be related to differences in study populations. Control hArg concentrations were much higher in the Yuan et al.^50^ study (3.31 vs. 5.4 μmol/L) and there were large difference in the BMI of the study participants. Future research on hArg concentrations throughout pregnancy, in women of varying BMIs and varying complications, such as PE, would be highly valuable.

Although strong cases for hArg's important, almost hormonal, role in pregnancy have been presented recently, very few studies have directly measured hArg concentrations in pregnancy complications (Table 2). The role of hArg in intrauterine growth restriction (IUGR), a complication closely linked to PE, has not been previously studied. IUGR occurs when a fetus does not grow at the expected rate for its gestational age, due to placental malformation and insufficiency. ADMA, NO and other arginine pathway metabolites in the maternal circulation have been associated with IUGR and small for gestational age (SGA) infants, but with varying findings^117^, ^118^, ^122^ (Table 2). Lower ADMA and arginine plasma concentrations have also been associated with pregnant women that were assessed with depressive symptoms^123^ and gestational diabetes, where insufficient insulin is produced during pregnancy.^124^, ^125^ Although hArg was not directly measured in these other studies, related compounds to hArg appear to play a role in these and other pregnancy‐related complications. Therefore, hArg should be included in future pregnancy complication studies, especially when measuring the concentrations of related compounds (ADMA, Arg, and NO). Such measurements would help to elucidate the mechanisms or altered pathways that may contribute to the pathogenesis of pregnancy complications.

## 5 CONCLUSIONS

**FIGURE 3:**
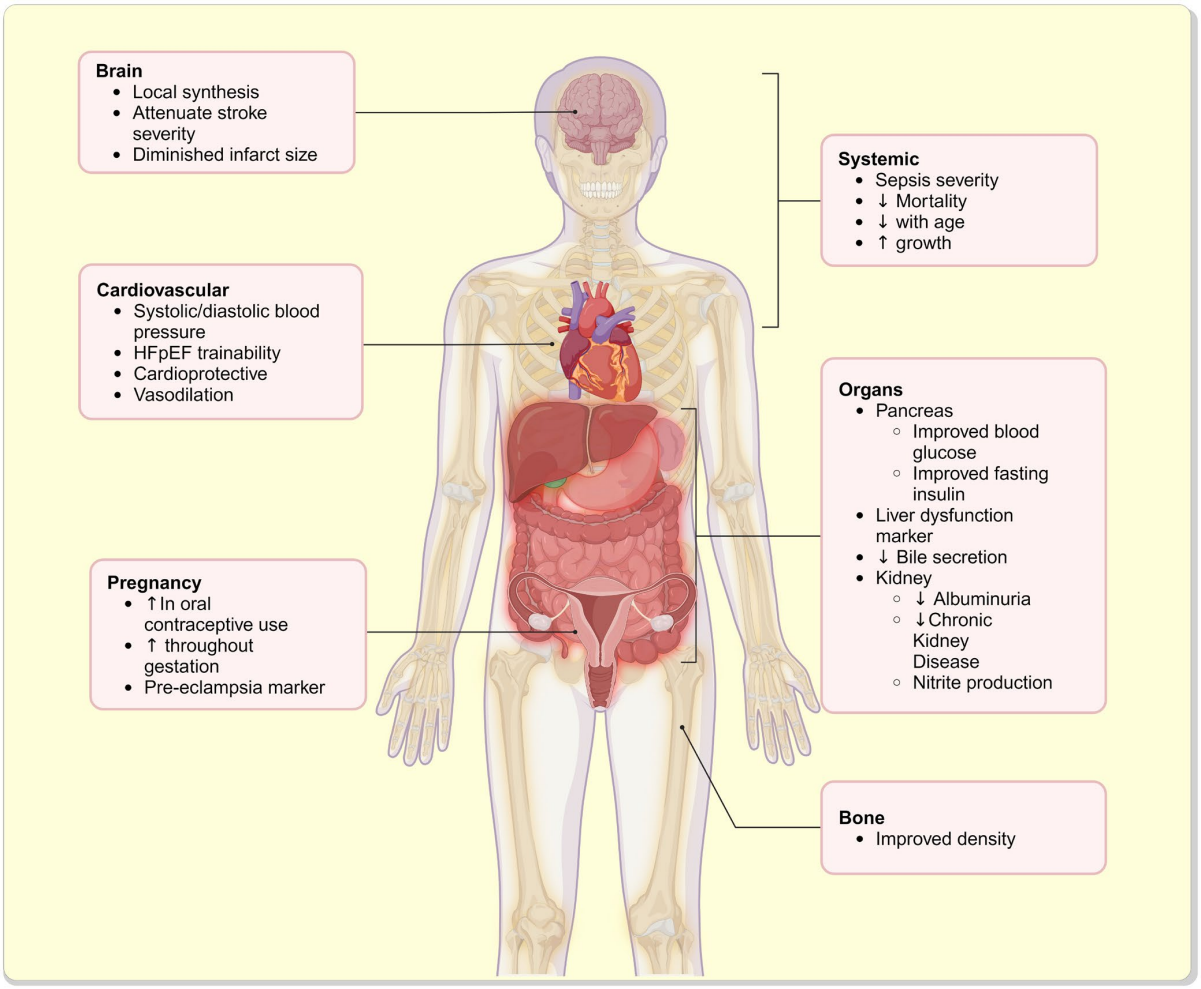
Extensive effects and responses of homoarginine (hArg) throughout the entire body system. HArg is synthesized in the brain, where it mitigates stroke severity and diminishes infarct size. The metabolite is recognized for its ability to promote vasodilation, enhance trainability of heart failure with preserved ejection fraction (HFpEF), and regulate blood pressure, creating a cardioprotective effect. Increased concentrations are found in oral contraceptive use and pregnancy, although its role in markers for pre‐eclampsia remains inconclusive. Systemically, hArg reduces mortality rates and can predict sepsis severity. Its concentration increases during periods of growth, and decreases with age. Various organs are affected by hArg, including the pancreas, liver, gallbladder and kidney. It also influences bone health. Created in BioRender. Wishart, D (2024). BioRender.com/z54q423 (#NU27EQO1TO).

HArg is an under‐appreciated amino acid that is synthesized by the AGAT and OTC enzymes via homologous biosynthetic mechanisms found for Arg. Arginase is a potential substrate and a confirmed inhibitor for hArg, while AGXT2 is known to degrade hArg for waste disposal. The few mechanistic studies on hArg that looked at enzyme/protein binding, gene regulation, or other pathways have shown that hArg can modulate nitric oxide (NO) production through its interaction with NOS.^33^, ^34^ Likewise, a number of studies have shown that hArg can control levels of Arg and Orn by inhibiting the action of arginase.^34^ Additionally, hArg can increase *Kcnip3* and *Ivd* gene expression,^35^ and bind/inhibit APs.^36^ Furthermore, hArg can bind to Myh9, which inhibits T‐cell activation and proliferation.^37^ Further exploration of these mechanisms will allow us to better understand hArg's role in human/animal pathophysiology and how hArg contributes to whole‐body homeostasis and disease mechanisms (Figure 3).

While mechanistic studies of hArg continue to provide key insights into its many physiological and pathophysiological roles, the exploitation of hArg as a potential disease biomarker for a growing list of conditions has continued. To date, most published hArg biomarker studies have examined its utility in assessing cardiovascular health. However, as seen in this review, hArg appears to be a useful biomarker for determining stroke outcomes, assessing CKD status, measuring sepsis severity, evaluating CNS conditions, as well as detecting or assessing diabetes, obesity, liver disease, bone density, pregnancy outcomes and pregnancy complications. In particular, low serum/plasma levels of hArg are associated with increased levels of endothelial dysfunction, more severe heart failure, greater cardiovascular mortality and poorer sepsis outcomes, while high serum/plasma levels of hArg are associated with inhibition of platelet aggregation, attenuation of stroke severity, hyperglycemia, abnormal levels of liver biomarkers and later pregnancy staging.

Although most hArg studies have focused on making health or disease associations, it is clear more mechanistic research is required to fully understand the role of hArg in the human body and why it functions as a biomarker for so many conditions. Those studies that have included more advanced metabolomic approaches and assessed multiple hArg‐related metabolites have played key roles in the enzymology of hArg metabolism. Using metabolomics in combination with pathway analysis and carefully measured in vitro responses to hArg could help further elucidate both hArg mechanisms and its underlying physiological effects. At this time, there is widespread agreement that hArg plays an important role in many cardiovascular and renal complications, but the pathogenesis of these complications and the mechanisms of hArg regulation still need to be established with further research.

## AUTHOR CONTRIBUTIONS

**Ashley Zubkowski:** Conceptualization; investigation; writing – original draft; writing – review and editing; data curation; formal analysis; visualization. **Amanda N. Sferruzzi‐Perri:** Conceptualization; writing – review and editing; writing – original draft; project administration; supervision; resources; funding acquisition; data curation. **David S. Wishart:** Conceptualization; funding acquisition; writing – original draft; writing – review and editing; project administration; supervision; resources; data curation.

## CONFLICT OF INTEREST STATEMENT

The authors declare that the research was conducted in the absence of any commercial or financial relationships that could be construed as a potential conflict of interest.

## Data Availability

Data sharing is not applicable to this article as no new data were created or analyzed in this study.
